# Supplementary material for: Climate drivers of plague epidemiology in British India, 1898–1949
Source: Proc Biol Sci. 2020 Jun 10;287(1928):20200538. doi: 10.1098/rspb.2020.0538 (PMC7341932; doi:10.1098/rspb.2020.0538)
Supplement: Supplementary Tables and Figures [file rspb20200538supp1.pdf]

# Climate drivers of plague epidemiology in British India, 1898–1949

Warren S. D. Tennant[1,3*]

Mike J. Tildesley[1,2,3]

Simon E. F. Spencer[1,4]

Matt J. Keeling[1,2,3]

[1]The Zeeman Institute: SBIDER, University of Warwick, Coventry, CV4 7AL

[2]School of Life Sciences, University of Warwick, Coventry, CV4 7AL

[3]Mathematics Institute, University of Warwick, Coventry, CV4 7AL

[4]Statistics Department, University of Warwick, Coventry, CV4 7AL

[*]Corresponding author: Warren.Tennant@warwick.ac.uk

Citation: Tennant, W. S. D., Tildesley, M. J., Spencer, E. F. S., and Keeling, M. J. (2020). Climate drivers of plague epidemiology in British India, 1898–1949. *Proc. R. Soc. B.* doi: 10.1098/rspb.2020.0538

## Supplementary material

| Bayes factor (BF) | Strength of evidence to support the alternative hypothesis |
|---|---|
| $< 1$ | Negative (supports the null hypothesis) |
| 1–3.2 | Weak |
| 3.2–10 | Substantial |
| 10–100 | Strong |
| $> 100$ | Decisive |

**Supplementary Table 1. Interpretation of Bayes factors.** Bayes factors were calculated for each regression model presented in the main manuscript. The table above, presented by Kass and Raftery (1995)[1], was used to assess the strength of evidence in support of each model over their respective null model (a model with no linear covariates) and their parameters being non-zero.

[1]Kass, R. E., & Raftery, A. E. (1995). Bayes Factors. Journal of the American Statistical Association, 90(430), 773. https://doi.org/10.2307/2291091

**A. Linear predictors and their 95% credible intervals.**

| Climate | Outbreak size | $\beta_0$ [95% CI] | $\beta_1$ [95% CI] | $\beta_2$ [95% CI] | $\beta_3$ [95% CI] |
|---|---|---|---|---|---|
| Temperature | 0 | 98.4 [70.9, 138.8] | -3.05 [-4.21, -2.23] | 0.023 [0.017, 0.032] | - |
| | 1 | 85.3 [60.8, 119.6] | -2.63 [-3.60, -1.90] | 0.020 [0.015, 0.027] | - |
| | 10 | 63.1 [47.4, 80.2] | -2.05 [-2.58, -1.55] | 0.016 [0.012, 0.020] | - |
| | 100 | 15.9 [4.16, 27.8] | -0.571 [-0.941, -0.206] | 0.005 [0.002, 0.008] | - |
| Precipitation | 0 | -4.11 [-5.43, -2.92] | 5.74 [4.60, 7.06] | -1.17 [-1.45, -0.925] | 0.064 [0.049, 0.081] |
| | 1 | -4.13 [-5.37, -2.99] | 5.66 [4.60, 6.82] | -1.22 [-1.47, -0.984] | 0.069 [0.055, 0.085] |
| | 10 | -5.32 [-6.66, -4.09] | 5.76 [4.74, 6.88] | -1.23 [-1.47, -1.01] | 0.070 [0.057, 0.086] |
| | 100 | -5.20 [-6.58, -3.95] | 4.90 [3.89, 6.02] | -1.13 [-1.39, -0.898] | 0.070 [0.055, 0.087] |
| Humidity | 0 | -70.1 [-54.3, -39.9] | 1.29 [1.72, 2.19] | -0.016 [-0.013, -0.010] | - |
| | 1 | -59.6 [-46.0, -33.4] | 1.10 [1.47, 1.88] | -0.014 [-0.011, -0.008] | - |
| | 10 | -69.0 [-55.5, -43.1] | 1.30 [1.71, 2.11] | -0.015 [-0.013, -0.010] | - |
| | 100 | -53.6 [-41.3, -29.9] | 0.91 [1.24, 1.59] | -0.012 [-0.009, -0.007] | - |

**B. Bayes factor for linear predictors and models.**

| Climate | Outbreak size | $BF_{\beta_0}$ | $BF_{\beta_1}$ | $BF_{\beta_2}$ | $BF_{\beta_3}$ | BF |
|---|---|---|---|---|---|---|
| Temperature | 0 | $> 1000$ | $> 1000$ | $> 1000$ | - | $> 1000$ |
| | 1 | $> 1000$ | $> 1000$ | $> 1000$ | - | $> 1000$ |
| | 10 | $> 1000$ | $> 1000$ | $> 1000$ | - | $> 1000$ |
| | 100 | 0.013 | 0.048 | 0.106 | - | 0.942 |
| Precipitation | 0 | $> 1000$ | $> 1000$ | $> 1000$ | $> 1000$ | $> 1000$ |
| | 1 | $> 1000$ | $> 1000$ | $> 1000$ | $> 1000$ | $> 1000$ |
| | 10 | $> 1000$ | $> 1000$ | $> 1000$ | $> 1000$ | $> 1000$ |
| | 100 | $> 1000$ | $> 1000$ | $> 1000$ | $> 1000$ | $> 1000$ |
| Humidity | 0 | $> 1000$ | $> 1000$ | $> 1000$ | - | $> 1000$ |
| | 1 | $> 1000$ | $> 1000$ | $> 1000$ | - | $> 1000$ |
| | 10 | $> 1000$ | $> 1000$ | $> 1000$ | - | $> 1000$ |
| | 100 | $> 1000$ | $> 1000$ | $> 1000$ | - | $> 1000$ |

**Supplementary Table 2. Effect of climate on outbreak occurrence.** The above tables present the results of the quadratic and cubic regression models investigating the effect of climate on outbreak occurrence. Different outbreak thresholds were tested. The median and 95% credible interval of each model parameter, the Bayes factor of the full model, $B$, and Bayes factor of each parameter being non-zero, $B_{\beta_i}$, are shown.

**A. Linear predictors and their 95% credible intervals.**

| Climate | $\beta_4$ [95% CI] | $\beta_5$ [95% CI] | $\beta_6$ [95% CI] | $\beta_7$ [95% CI] |
|---|---|---|---|---|
| Temperature | 3.64 [-3.24, 10.6] | -0.075 [-0.295, 0.141] | 0.001 [-0.001, 0.002] | - |
| Precipitation | 0.483 [-0.058, 1.02] | 1.57 [1.20, 1.95] | -0.378 [-0.455, -0.301] | 0.023 [0.018, 0.027] |
| Humidity | -19.2 [-24.6, -13.7] | 0.650 [0.491, 0.809] | -0.005 [-0.006, -0.004] | - |

**B. Bayes factor for linear predictors and models.**

| Climate | $\text{BF}_{\beta_4}$ | $\text{BF}_{\beta_5}$ | $\text{BF}_{\beta_6}$ | $\text{BF}_{\beta_7}$ | BF |
|---|---|---|---|---|---|
| Temperature | $< 0.001$ | $< 0.001$ | $< 0.001$ | - | $< 0.001$ |
| Precipitation | 0.002 | $> 1000$ | $> 1000$ | $> 1000$ | $> 1000$ |
| Humidity | 514 | $> 1000$ | $> 1000$ | - | $> 1000$ |

**Supplementary Table 3. Effect of climate on outbreak magnitude.** The above tables present the results of quadratic and cubic regression models investigating the effect of climate on outbreak magnitude. The median and 95% credible interval of each model parameter, the Bayes factor of the full model, $B$, and Bayes factor of each parameter being non-zero, $B_{\beta_i}$, are shown.

**A. Linear predictors and their 95% credible intervals.**

| Climate | Location control | $\beta_8$ [95% CI] | $\beta_9$ [95% CI] |
|---|---|---|---|
| Temperature | No | -12.56 [-14.2, -11.0] | 1.82 [1.59, 2.06] |
| Precipitation | No | 7.06 [6.09, 8.02] | -1.29 [-1.45, -1.12] |
| Humidity | No | -3.83 [-4.37, -3.28] | 1.05 [0.896, 1.20] |
| Temperature | Yes | 0.001 [-0.039, 0.042] | 0.198 [-0.013, 0.411] |
| Precipitation | Yes | 0.001 [-0.040, 0.042] | 0.095 [-0.050, 0.239] |
| Humidity | Yes | 0.002 [-0.042, 0.044] | 0.804 [0.585, 1.03] |

**B. Bayes factor for linear predictors and models.**

| Climate | Location control | $\text{BF}_{\beta_8}$ | $\text{BF}_{\beta_9}$ | BF |
|---|---|---|---|---|
| Temperature | No | $> 1000$ | $> 1000$ | $> 1000$ |
| Precipitation | No | $> 1000$ | $> 1000$ | $> 1000$ |
| Humidity | No | $> 1000$ | $> 1000$ | $> 1000$ |
| Temperature | Yes | $< 0.001$ | 0.19 | 0.18 |
| Precipitation | Yes | 0.001 | 0.05 | 0.05 |
| Humidity | Yes | $< 0.001$ | $> 1000$ | $> 1000$ |

**Supplementary Table 4. Effect of climate on outbreak timing.** The above tables present the results of the linear regression models investigating the effect of climate on outbreak timing. The median and 95% credible interval of each model parameter, the Bayes factor of the full model, $B$, and Bayes factor of each parameter being non-zero, $B_{\beta_i}$, are shown.

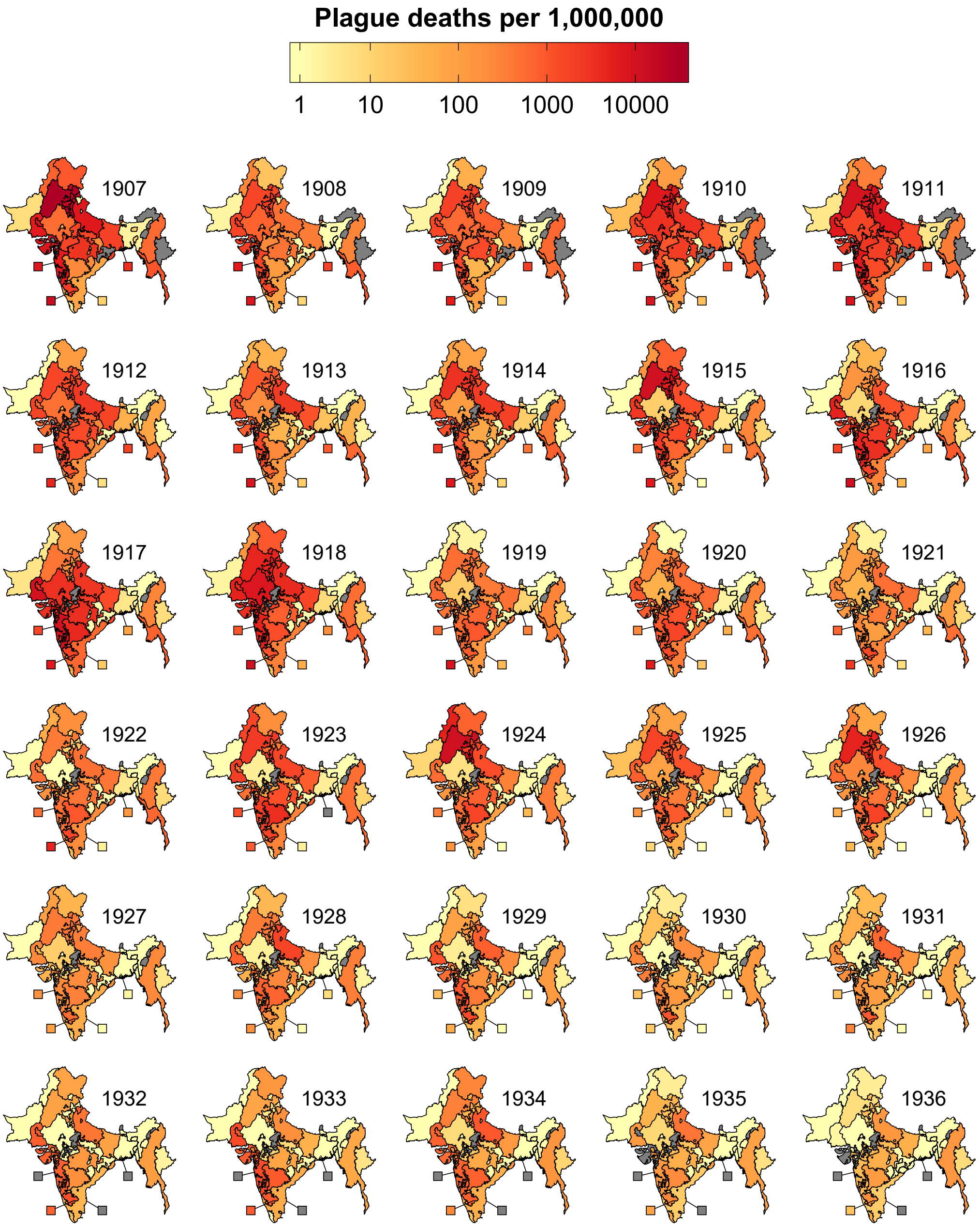


**Supplementary Figure 1. Annual plague deaths per 1,000,000 in British India from 1907–1936.** The total number of plague deaths per province, month and political division from 1907–1936 were taken from the annual Chief Indian Commissioner reports. There was a steady decrease in absolute mortality over time with a high degree of spatial heterogeneity across British India. Grey provinces represent the absence of plague data.

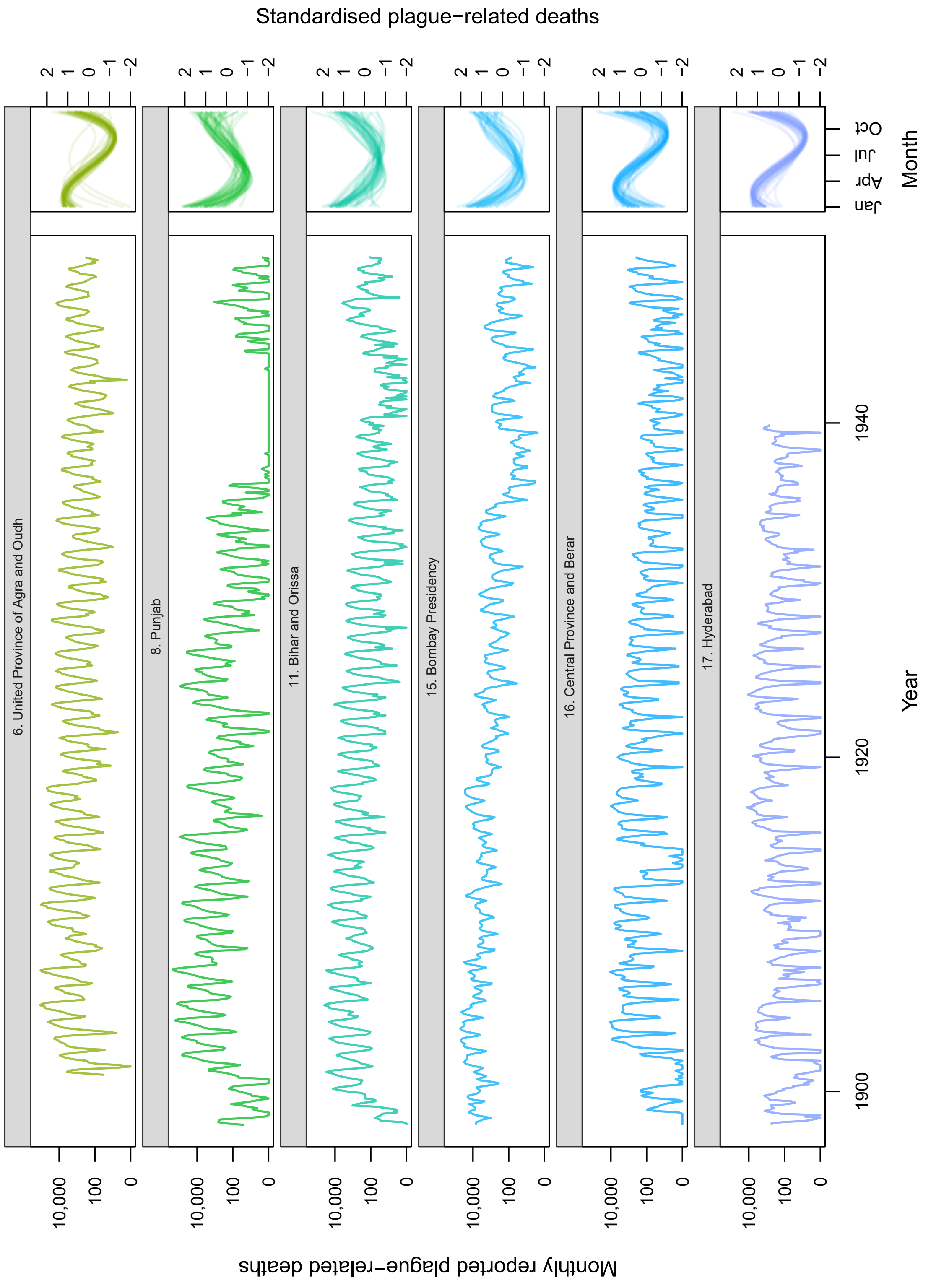


**Supplementary Figure 2. Plague deaths per province in British India from 1898–1949.** The total number of reported plague deaths per political division in provinces with the six highest number of reported cases from 1898–1949. Plague would emerge annually within these provinces with frequent reported of over 10,000 cases at the peak of each outbreak.

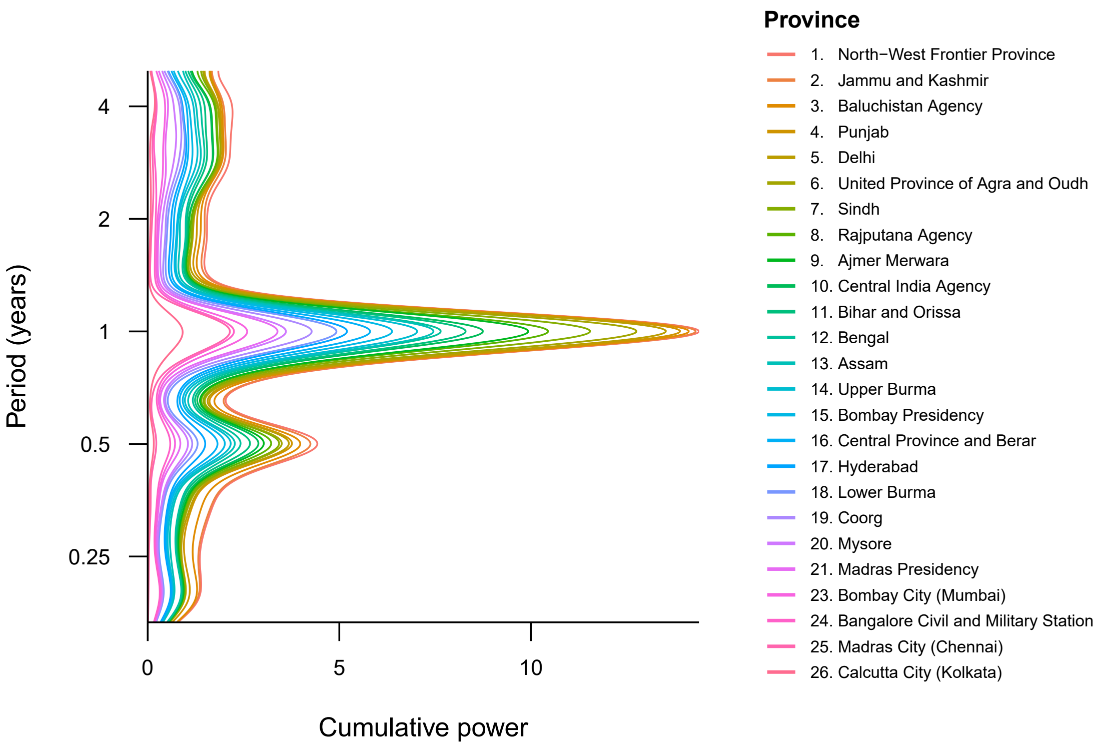


**Supplementary Figure 3. Periodicity of plague cases in British India from 1898–1949.** Wavelet analysis was performed on plague cases from each province in British India from 1898–1949. There was strong evidence for annual, and less-so for six-monthly, seasonal behaviour of plague outbreaks. The power was calculated from the average power of each period in the Wavelet Power Spectrum for each province.

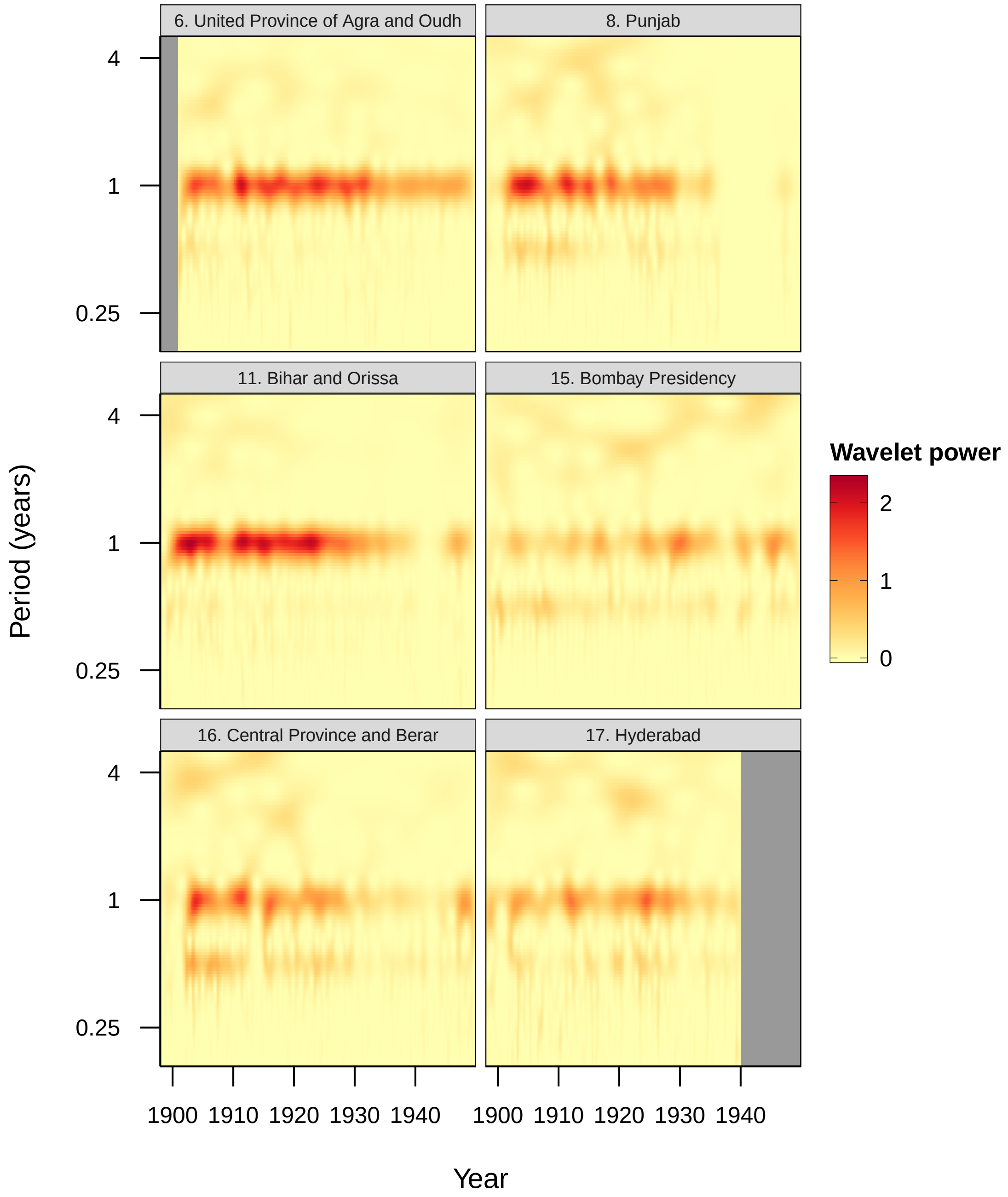


**Supplementary Figure 4. Wavelet Power Spectrum of plague cases in most affected provinces in British India from 1898–1949.** Each province shows high power at periods of one year. Some provinces show weaker powers, but time consistent, powers for periods of six months. The Wavelet Power Spectrum was calculated from the $\log_{10}$-transformed plague incidence data using the `WaveletComp` package in `R`.

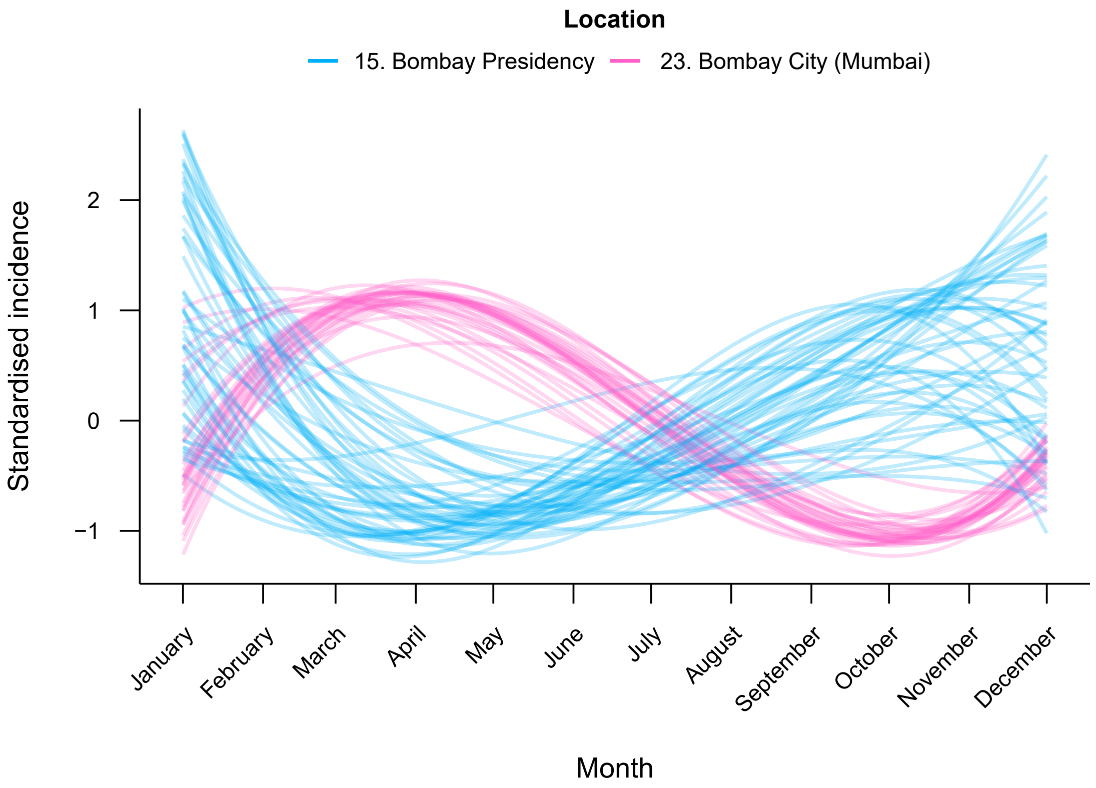


**Supplementary Figure 5. Asynchrony of outbreaks between Bombay City (Mumbai) and Bombay Presidency.** Incidence of plague mortality for Bombay Presidency and Bombay City (Mumbai) was normalised for each province and year to show relative outbreak timing. The timing of annual outbreaks in Bombay Presidency were out of synchronisation with outbreaks within Bombay City. Outbreaks across Bombay Presidency were also sustained for a longer period of time compared with Bombay City.

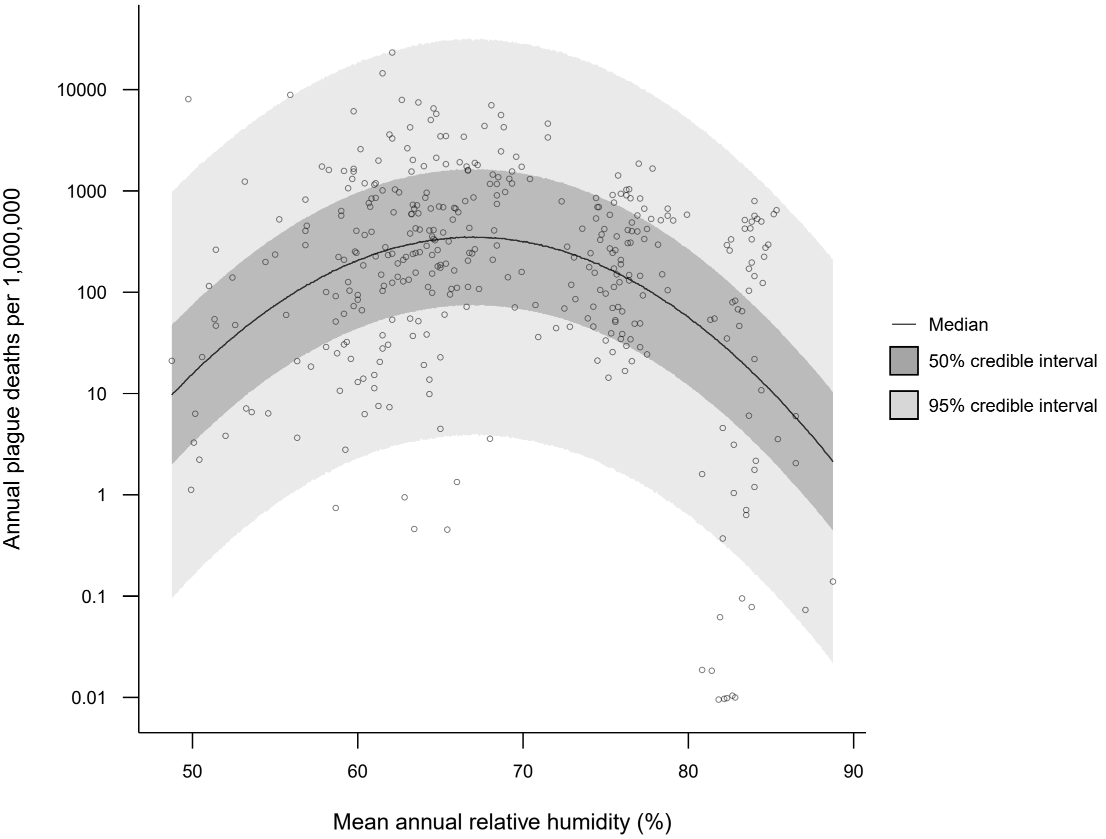


**Supplementary Figure 6. Effects of humidity on outbreak magnitude.** Annual outbreaks were larger for moderate humidity values, compared with lower and higher relative humidity. However, although a quadratic relationship between humidity and outbreak magnitude was maintained ($B > 1000$), humidity alone was not enough to precisely predict the size of each outbreak. Credible intervals were generated from fitting a cubic polynomial to incidence data on the log-scale with weakly informative Cauchy priors on linear predictors and assuming Gaussian error structure of the residuals using the `R` package `rstanarm`.

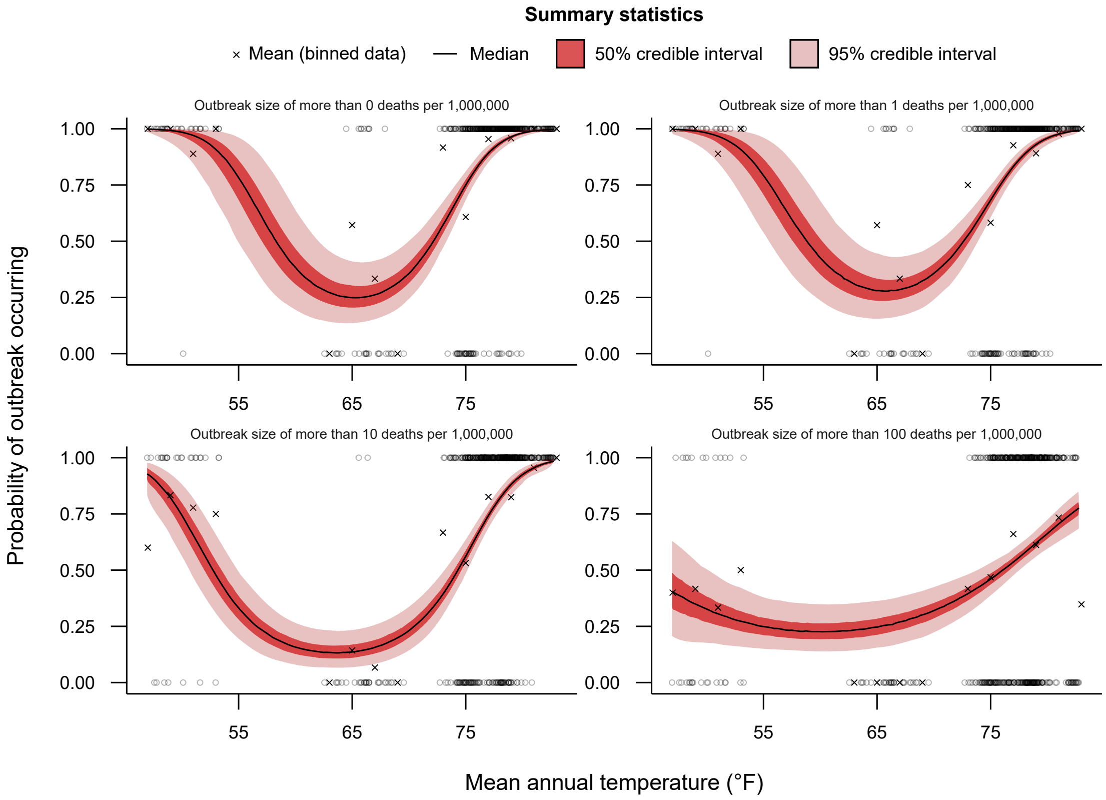


**Supplementary Figure 7. Temperature effects on outbreak occurrence**. Outbreaks had a higher probability of occurring at low and high levels of mean annual temperature. At moderate temperatures, outbreaks of any size rarely occurred ($B > 1000$). However, this relationship was not consistent across outbreak sizes, with no evidence to suggest a relationship between temperature and outbreak occurrence with an outbreak threshold of over 100 deaths per 100,000 individuals ($B < 0.001$). Credible intervals were calculated from fitting the mean humidity per year per province to whether outbreaks occurred or not under different thresholds for defining an outbreak. Circles denote the binary data that was fit, and crosses show the mean probability of an outbreak occurring at binned humidity values. A quadratic polynomial was fit within a Bayesian framework with weakly informative Cauchy priors on coefficients and assumed binomial error structure using the `rstanarm` package in `R`.

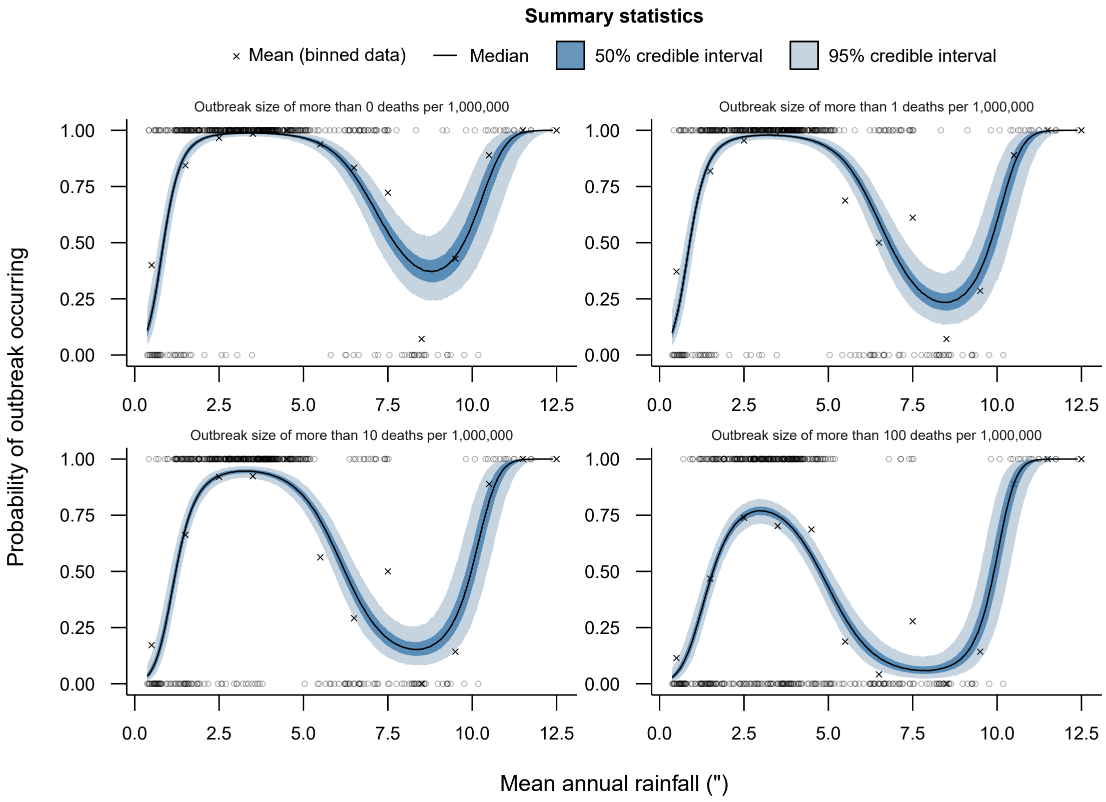


**Supplementary Figure 8. Rainfall effects on outbreak occurrence**. Outbreaks had a higher probability of occurring at moderately low and high levels of mean annual rainfall. At low and moderate rainfall levels, outbreaks of any size rarely occurred ($B > 1000$). Credible intervals were calculated from fitting the mean humidity per year per province to whether outbreaks occurred or not under different thresholds for defining an outbreak. Circles denote the binary data that was fit, and crosses show the mean probability of an outbreak occurring at binned humidity values. A cubic polynomial was fit within a Bayesian framework with weakly informative Cauchy priors on coefficients and assumed binomial error structure using the `rstanarm` package in `R`.

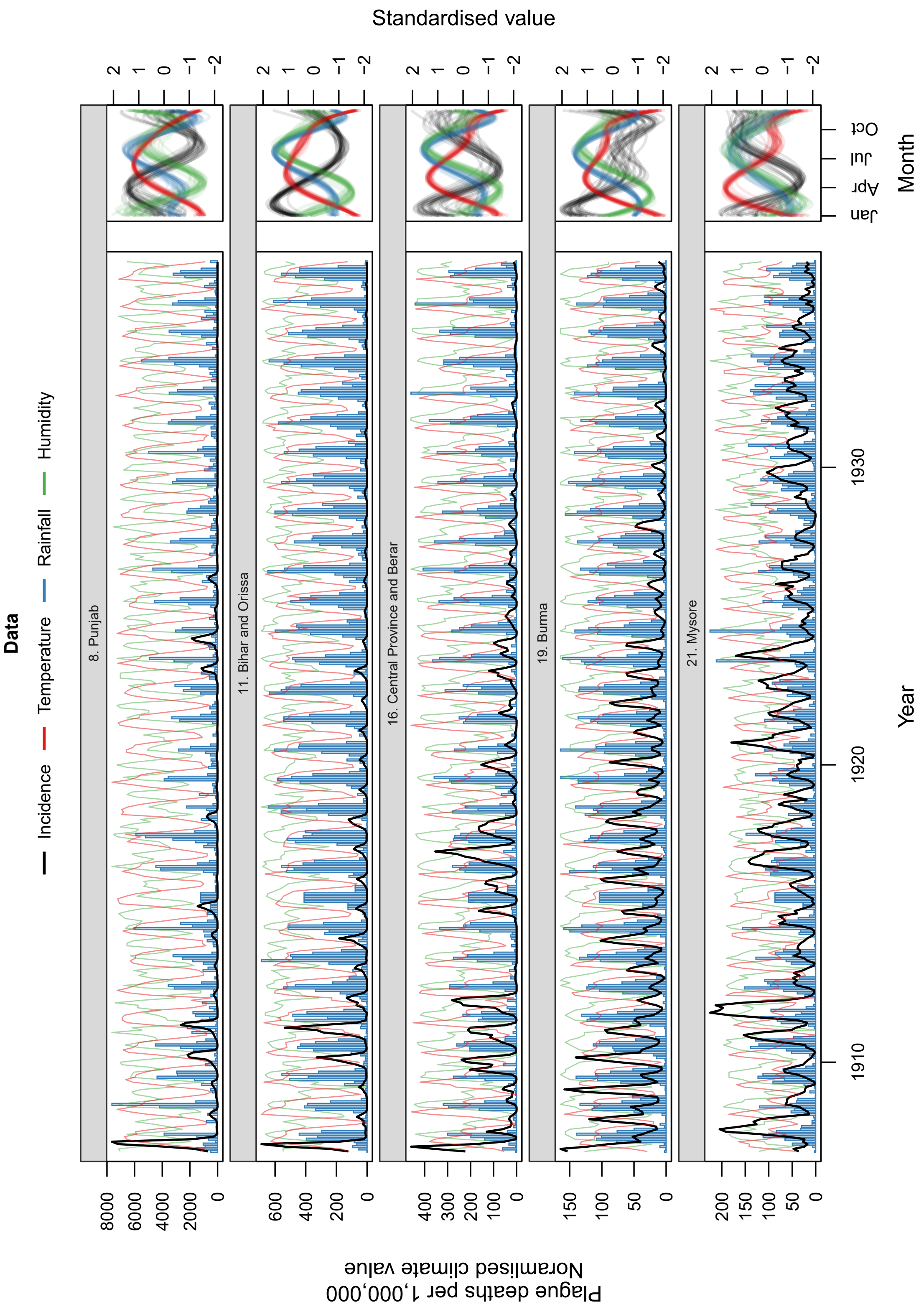


**Supplementary Figure 9. Climate throughout British India from 1907–1936.** Temperature, humidity and rainfall data were collected for each province in British India from 1907–1936. This data were not complete, so plague data were aggregated to a similar spatial scale found in the climate data. For example, incidence data for Lower and Upper Burma were combined to allow comparison with humidity data found on the same level. Here, for the purposes of visualisation, temperature and rainfall data for Lower and Upper Burma were also aggregated.

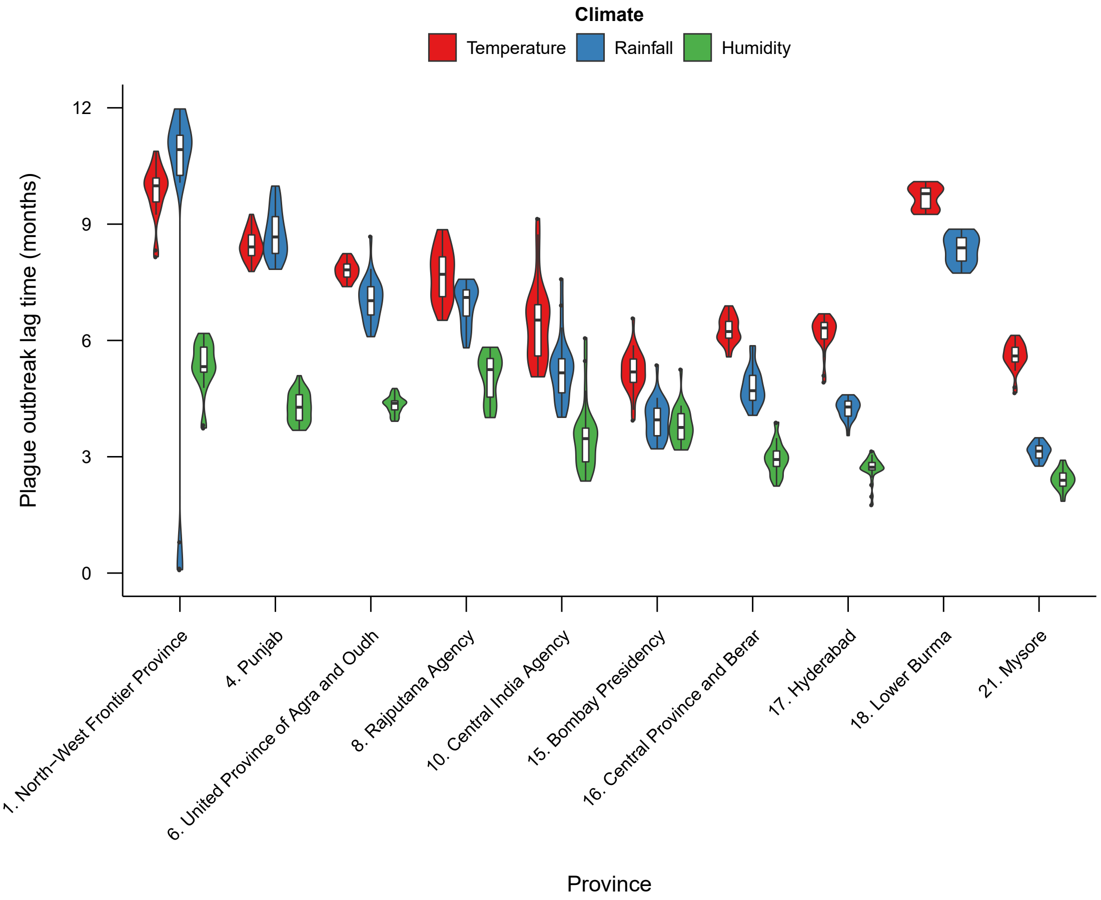


**Supplementary Figure 10. Variation in lags between plague outbreaks and climate.** The time between plague outbreaks and seasonal oscillations in temperature, rainfall and humidity were found to be largely consistent across time in each province. Lag times were only calculated in the year proceeding when a plague outbreak occurred. In general, lag times between temperature and plague outbreaks were the longest, with humidity being the shortest. Lag times associated with all climate variables also increased from the south (Mysore) to the north (North-West Frontier Province).

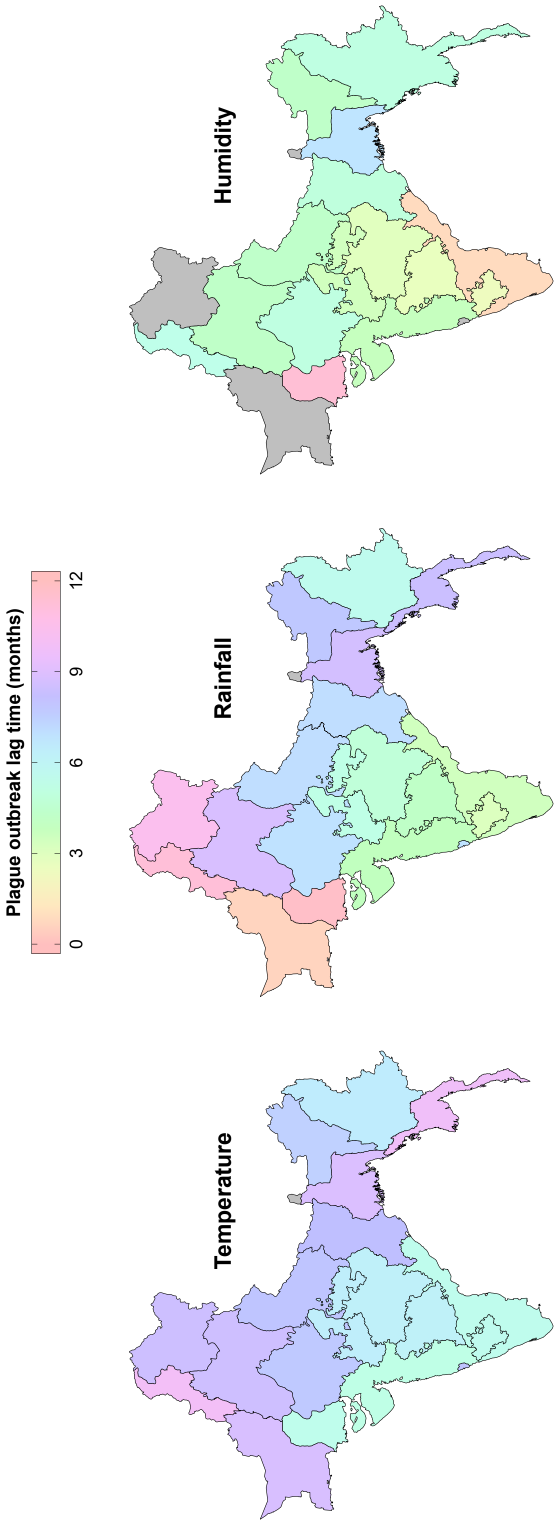


**Supplementary Figure 11. Spatial variation in lags between plague outbreaks and climate.** Time lags between plague outbreaks and seasonal oscillations in temperature, rainfall and humidity were not consistent between different provinces. However, lags did exhibit a similar south-to-north pattern as the timing of plague outbreaks, suggesting that climate only accounts for some variation in the timing of outbreaks across different provinces. Over half the outbreaks had delays of 6–9 months, 4–8 months and 3–5 months with temperature, rainfall and humidity, respectively.

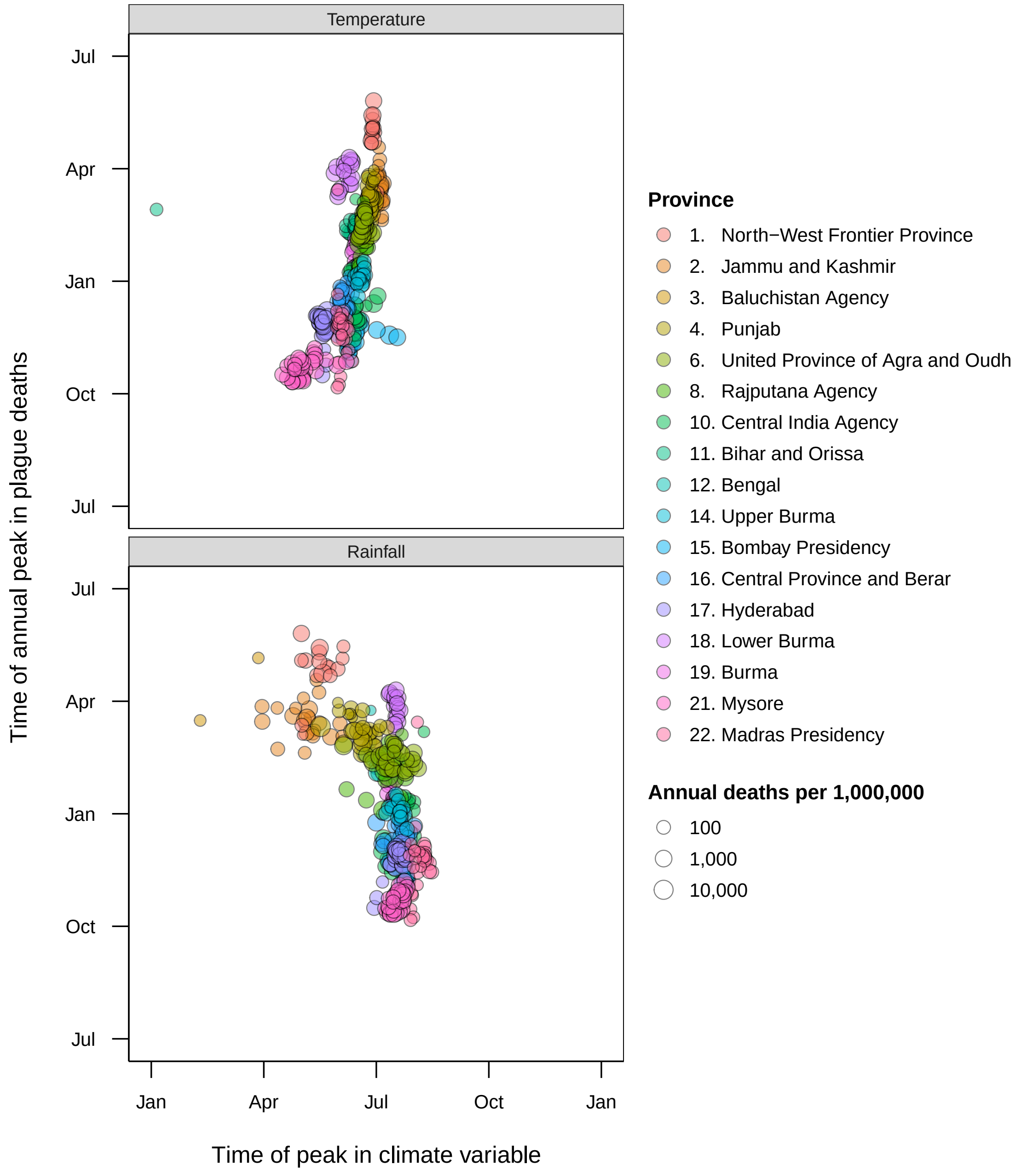


**Supplementary Figure 12. Climate against plague outbreak timing.** There was a strong relationship between the timing of oscillations in rainfall and plague outbreaks ($B > 1000$), where later peaks in rainfall were associated with earlier outbreaks. In contrast, there was a positive linear correlation between humidity and the timing of outbreaks at the national scale: outbreaks occurred the later humidity peaked. There was little evidence to suggest a correlation between temperature and outbreak timing because there little time-variation in the oscillations of temperature between different provinces.

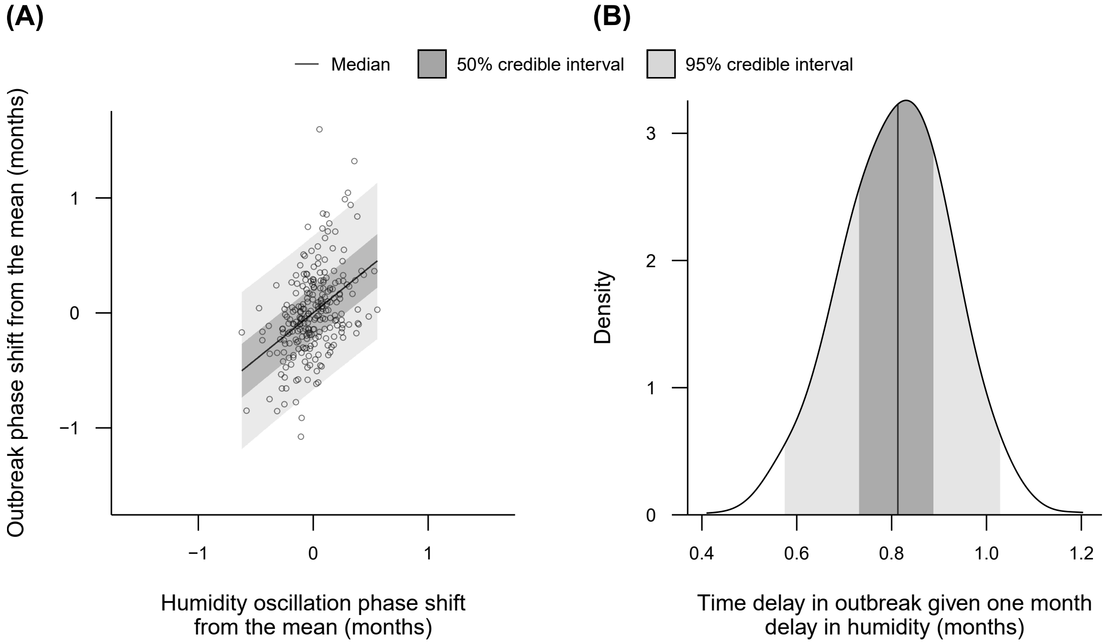


**Supplementary Figure 13. A delay in humidity delays outbreaks.** After correcting for the timing of outbreaks and climate within each province, **(A)** the linear relationship between the timing of outbreaks and oscillations in humidity was maintained. **(B)** On average, for every one month that seasonal changes in humidity were delayed, plague outbreaks would occur approximately 3.5 weeks later. Credible intervals were generated from fitting a linear model to the data within a Bayesian framework with weakly informative Cauchy priors on linear predictors and assumed Gaussian error structure.

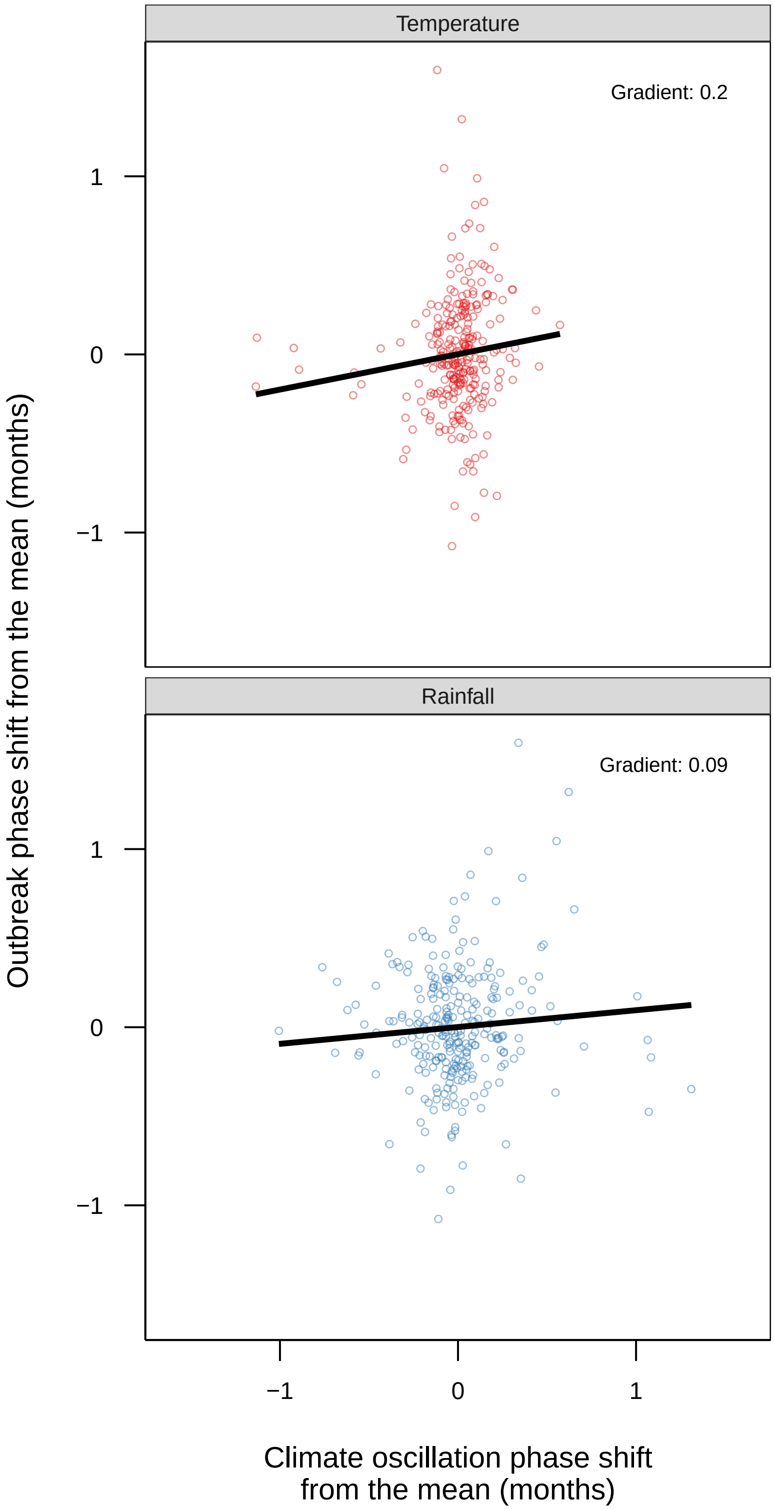


**Supplementary Figure 14. Temperature and rainfall only have a small influence on outbreak timing.** By observing changes in the timing of each climate variable from the normal against the equivalent for plague outbreaks, we found there to be no evidence to support a relationship between seasonal changes in temperature and rainfall with the timing of outbreaks ($B < 0.001$ and $B < 0.001$, respectively). On average, a delay of one month in the timing of temperature and rainfall resulted in a delay of only 6 and 3 days in plague outbreaks respectively.

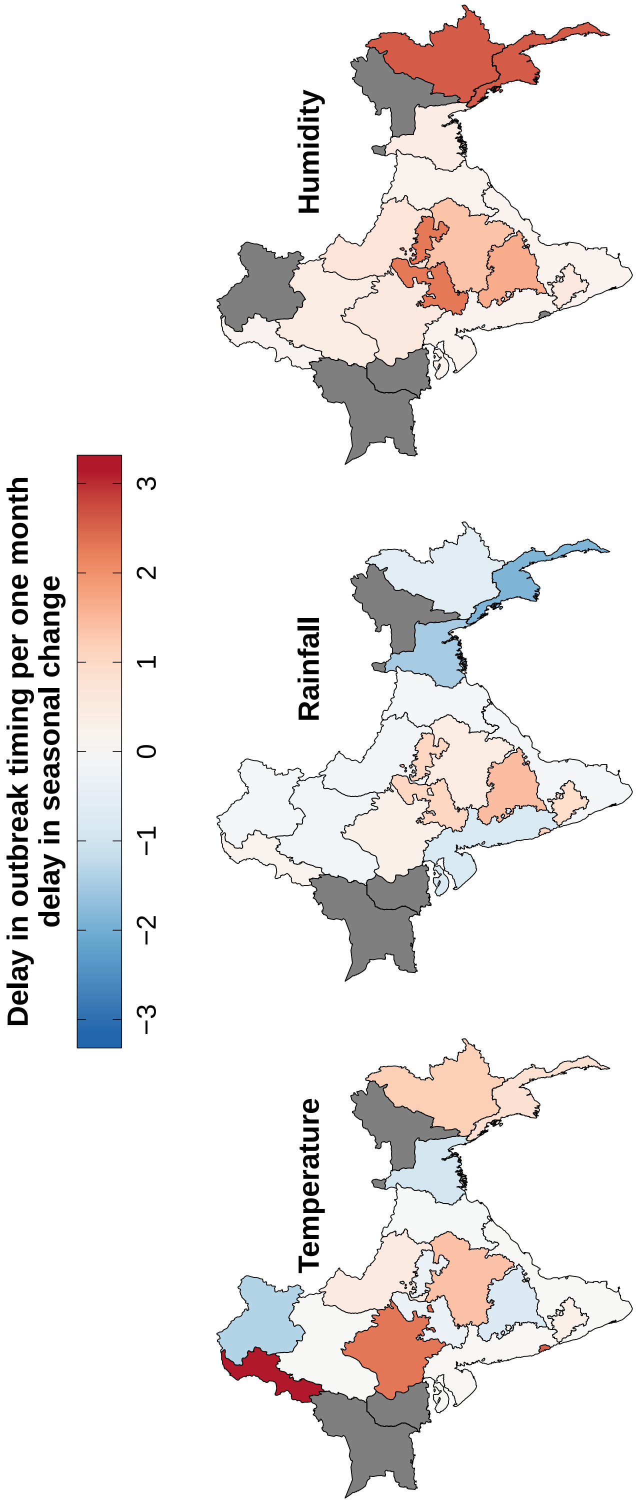


**Supplementary Figure 15. Provincial variation of the influence of climate on outbreak timing.** These maps show the time that plague outbreaks were delayed given a single month delay in seasonal changes for temperature, rainfall and humidity. There was a high degree of variation in delay time between provinces for each climate variable. All delays for humidity were positive.
